# Policy implications for improved and equitable mental health – the Swedish case: a summative content analysis of findings across 26 years from two qualitative meta-syntheses

**DOI:** 10.1186/s12889-026-27673-x

**Published:** 2026-06-02

**Authors:** Anne Hammarström, Per-Olof Östergren, Gunnar Ågren, Urban Janlert, Hugo Westerlund, Maria Albin

**Affiliations:** 1 Institute of Environmental Medicine, Karolinska Institutet, Nobels väg 13, Solna, 171 65 Sweden; 2 Department of Epidemiology and Global Health, Umeå University, Umeå, SE 90187 Sweden; 3 Social Medicine and Global Health, Department of Clinical Sciences in Malmö, Lund University, CRC Malmö, Malmö, SE 20205 Sweden; 4 Former Swedish National Institute of Public Health Götgatan 83E, Stockholm, 11662 Sweden; 5 Stress Research Institute, Department of Psychology, Stockholm University, Stockholm, SE-106 91 Sweden

**Keywords:** Qualitative methods, health policies, social determinants of health, life-course epidemiology, unemployment, job insecurity, working conditions, gender relations, social support, residential characteristic

## Abstract

**Background:**

Policy implications are seldom more than a paragraph or two at the end of empirical papers. The aim was therefore to explore social determinants of mental health across life through qualitative analysis of meta-synthesis of empirical publications from a 27-year school-leaver cohort. From the actionable empirical results, we suggest policies for primary prevention aimed at equitable mental health across life.

**Methods:**

Empirical data was taken from two qualitative meta-syntheses which integrate findings from 79 empirical papers based on a longstanding Swedish school-leaver study about determinants of mental health during life. Summative content analysis guided our approach to further synthesize the findings from the meta-syntheses into actionable key themes.

**Results:**

We suggest possible contextualised polices based on the following ten key themes: (1) Unemployment can cause poor mental health. (2) Active labour market programs may counteract poor mental health. (3) Temporary employment is detrimental to mental health. (4) Good school climate could be protective against later mental health symptoms. (5) Poor working conditions are related to poor mental health. (6) Material and social adversities can cause poor mental health. (7) Gender inequities in working life are related to poor mental health. (8) Gender inequities in private life are related to poor mental health. (9) Poor social support is detrimental to mental health. (10) Disadvantaged neighbourhood seem to have a weak but consistently negative impact on mental health.

**Conclusion:**

Our qualitative analyses of cohort data, with a broad life-course approach, synthesised actionable results from which we identified evidence-based policies for improved and equitable mental health from adolescence until midlife. We encourage further systematisation of evidence-based public health policies in other contexts, including the ongoing marketisation and underfunding of the welfare sector in high-income countries.

Funding supporting health equities and allocations by needs should be regarded as investments in sustainability.

## Background

Mental health problems are multifactorial with social, behavioural as well as biological (including genetic) determinants, which interact in a complex manner [1] For population-level prevention, more scientifically rigorous knowledge about social determinants of mental health is needed [2]. While mental health disorders should be clinically diagnosed, we here focus on mental health symptoms reported by individuals using validated self-completion scales. In this paper, we address the social determinants of mental health, based on high-quality longitudinal studies from youth until middle age. We present an example of the evolvement of a Nordic welfare state in relation to inequities in mental health. The study starts in the early 1980s, when Sweden was one of Europe’s most developed welfare states with a relatively equal distribution of income and low inequities in health, and continues across four decades with major shifts in policies and rapidly increasing inequities in both income and health [3, 4]. During this period, the government monopoly on welfare distribution was challenged by the introduction of pseudo-market principles for the allocation of welfare services in several policy areas including health care, education and labour market policy. The context of the paper is mainly relevant to high income countries, particularly those employing neoliberal policies.

Over the past decades, mental health problems have increased considerable among young people. This increase is seen both in self-reported symptoms of depressiveness, anxiety and functional somatic symptom) [5] and in child psychiatric diagnoses [6]. These and similar results from around the world have led the Lancet Psychiatric Commission to conclude that the increase of youth mental health problems is a crisis of global importance [7]. The commission elaborates on global megatrends which may influence these problems, such as social media and climate change. Another mentioned megatrend is neoliberalism which for four decades has resulted in precarity in the job sector with increased insecurity and generational inequalities [7].

The social determinants of mental health, including the well-known class gradient in mental health [8] makes it necessary to move from merely treating individuals to addressing the determinants of population mental health with broader strategies [9]. Researchers need to contribute to evidence-based and effective social policies grounded in their empirical research. Policy implications are, however, seldom more than a paragraph or two at the end of empirical papers.

The objective of this study was therefore to extract evidence from long-term cohort data to identify policies for achieving equitable mental health from adolescence until midlife. The aim was to explore social determinants of mental health across life through qualitative analyses of meta-synthesis of earlier, empirical publications from a 27-year school-leaver study. Based on the actionable empirical results, we suggest policies for primary prevention aimed at equitable mental health across life.

## Methods

### Material

The data was drawn from earlier analyses of research papers based on the Northern Swedish Cohort (NoSCo), a 27-year longitudinal school leaver study starting at age 16 in 1981 in a municipality in Northern Sweden. The cohort has been followed regularly with questionnaires and health examinations. The participation rate has been extremely high, 94.3% (1010 out of 1071) at age 43. Except for being exposed to a higher rate of unemployment during the 1980s, the cohort is nationally representative [10].

The material for this study consisted of two qualitative meta-syntheses and the 82 empirical publications which they were based on. The publications were derived from an extensive research programme about determinants of mental health during life and included all international referee-reviewed publications (55 related to working-life [11], 27 related to the non-work sphere [12]) of NoSCo with mental health as outcome. Three of the 55 publications related to working-life were excluded due to cross-sectional design. In total, 79 publications were re-analysed together with empirical analyses of two qualitative meta-syntheses for this paper.

### Outcome

A validated, composite measure of self-reported symptoms of mental health was used as outcome in the empirical papers, consisting of symptoms of depressiveness, anxiousness and/or functional somatic symptoms [13].

All papers controlled for socioeconomic conditions at age 16. At the time when the study was initiated it was not considered ethically accepted to ask for childhood maltreatment or abuse, so we lack quantitative data on this important topic. In addition, the publications do not include data about living conditions before age 16.

The 79 publications have been analysed in the meta-syntheses, using the well-known method of analysing texts, qualitative content analysis for interpreting the content [14]. Meaning units (a constellation of words or sentences which relate to the same meaning) were identified, condensed and coded into categories and subthemes. The concept “categories” (answering the question “what”) was used in one of the meta-synthesis [11] while the concept “subthemes” was used in the other [12]. The final stage of analyses in each meta-synthesis was “theme”, answering the question “how” [14]. In total, 14 themes (with their 54 categories, 16 subthemes and 86 meaning units, respectively) were identified from the two meta-syntheses and were, together with the 79 original publications, the main material for this paper.

### Analyses

Our analysis was guided by a qualitative method called summative content analysis [15] which involves counting, comparisons and interpretation of the main content. The method was used to interpret meaning from the content of text data. To minimise bias, the selection of publications and their coding into themes have been carefully described. We performed the analyses in the following steps. First, all coded material from the two meta-analyses were analysed and compared. Also, all earlie coded material from the empirical publications, which the meta-analyses were based on, were reanalysed. AH did the first preliminary coding of all material, which was discussed among the authors in a collective process. We counted and compared the material, interpreted and synthesised it into key themes. Thus, the coding of all material was made in a systematic and careful way by several of the authors. Finally, we further developed our findings into policies for primary prevention.

Table 1 shows the original themes (and when needed also their sub-coding) and how we reanalysed them into new key themes. Of the 14 original themes, nine were synthesised and partly renamed into key themes. One abstract original theme was divided into its categories to make the results more concrete and possible to link to specific policy contexts. Also, one policy-relevant subtheme (Good school climate) was developed into a key theme of its own. Five original themes (of which two were similar) were excluded, as explained in Table 1. Of the remaining themes, two were similar and merged into one new key theme. In total, we identified 10 key themes for this paper and used them to derive possible policy interventions from for improved mental health during life (see Results and Table 2). Table 1 refers to coding process in more detail.

**Table 1:** Summative content analyses of themes, subthemes, categories and meaning units from the two meta-syntheses [11, 12] into key themes of this paper (references to the meta-synthesis are given in both columns)

Original themes from two meta-synthesis	Summative content analyses into new key themes
“Macroeconomic recession impairs mental health among young people.” [11]	Excluded, as the two included studies did not control for baseline mental health.
“The mental health effects on individuals of youth unemployment seem rather insensitive to recession.” [11]	Included in key theme 1: “Unemployment can cause poor health – both direct and long-term effects.” [11]
“Small but consistent negative effect of neighbourhood unemployment and other work-related disadvantaged on individuals’ mental health over life.” [11]	Developed into key theme 10: “Disadvantaged neighbourhood seem to have a weak but constant negative impact on mental health over life.” [11, 12]
“Youth unemployment becomes embodied as scars of mental ill-health over life.” [11]	Developed into key theme 1: “Unemployment can cause poor mental health – both direct and long-term effects.” [11]
“Weak LMA impairs mental health over life.” [11]	Divided into key theme 3: “Temporary employment is detrimental to mental health.” [11] and into key theme 1 above “Unemployment can cause poor mental health– both direct and long-term effects.” [11]
“ Bidirectional relations between health and weak LMA over life.” [11]	Included in the following key themes “1. Unemployment can cause poor mental health– both direct and long-term effects.” [11] and “2. Active labour market programs counteract poor health in the risk group of weak LMA.” [11] and “3. Temporary employment is detrimental to mental health.” [11]
“Macrolevel structures are of importance for how labour market position can cause poor health.” [11]	The theme was too vague and therefore divided into its categories to make them more concrete and possible to direct policies towards. Two categories (“Participation in youth opportunity programs was beneficial for health among teenagers.^”4^ And “Active labour market policies directed towards youths could potentially reduce the short- and long-term mental health costs of youth unemployment.”^4^) were developed into key theme 2: “Active labour market programs counteract poor health in the risk group of weak LMA.^”4^
	The categories related to “Accumulated unemployment [11] as well as the category “Physical heavy work could explain the effect of the socio-economic gradient on mental health in young adulthood.” [11] were developed into key themes 5: “Poor working conditions can cause poor mental health.” [11] and 1: “Unemployment can cause poor health – both direct and long-term effects.” [11]
	The categories “Growing up in an unfavourable socioeconomic circumstance can initiate a chain of risk of adverse living condition, which in turn affects adult health”, “Early adversities modify the relation between job characteristics and biological stress” [12] and “No mental health selection into class-related musculoskeletal disease” [12] as well as “No health selection into social mobility” [12] were developed into key theme 6: “Early and accumulated social and material adversities can cause poor mental health over life.” [12]
“Unequal gender relations at various levels impact negatively on mental health.” [12]	Developed into key theme 7: “Gender inequities in working life are related to poor mental health.” [12] And into key theme 8: “Gender inequities in private life are related to poor mental health.” [12]
“The agency to improve health over life in dyadic relations.” [11]	Excluded due to lack of analysis of social determinants of health.
”Relation between neighborhood disadvantage and mental ill-health over the life-course.” [12]	Developed into key theme 10: “Disadvantaged neighbourhood seem to have a weak but constant negative impact on mental health over life.” [11, 12]
“Relation between parental interaction with other settings and mental health over the life-course.” [12]	Included into key theme 9: “Poor social support could be detrimental to mental health over life.” [12]
“Relation between poor social relationships and mental ill-health over the life-course.” [12]	One subtheme “Good school connectiveness in early life was related to later mental health” [12] was developed into key theme 4: “Good school climate could be protective for later mental health symptoms.” [12] The other five categories were included in key theme 9: “Poor social support is detrimental to mental health over life.” [12]
“Relation between adversities and the socio-economic gradient of mental ill-health during life.” [12]	Developed into key theme 6: “Material and social adversities can cause mental ill-health.” [12]
“Relation between gender relations and improved mental health in adult life.” [12]	Developed into key theme 7: “Gender equity in adult life is related to good mental health.” [12]

**Table 2:** Empirical findings (presented as key themes) and suggested policy implications at various levels (N=National, M= Municipality)

Domains and Key themes	Policy implications
WORKING LIFE including school climate	
1. Unemployment can cause poor health – both direct and long-term effects. 2. Active labour market programs (ALMP) counteracts poor health in the risk group of weak LMA.	Goals: Combat unemployment. Decrease the negative consequences of unemployment. Suggested interventions: N: To create more jobs, several policies can be applied including reduction of working hours and overtime work. Also, a shift between taxes on wages and on capital has been suggested [16]. N, M: Educational policies addressing mismatch between labour supply and demands. N, M: Social and psychological support and rehabilitation when needed. N: Expansion of subsidised employment schemes for those furthest from the labour market. N, M: Active labour market programs, especially for young people in NEET. N: Sufficient financial compensation during unemployment. In addition, financial support would be valuable for those in need of further vocational education. N: Improve possibilities for all employees to keep their protection from the employment protection act while testing a new job.
3. Temporary employment is detrimental to mental health	Goals: Promote secure employment contracts Improve access to and coverage of welfare benefits which already are linked to standard employment. Suggested interventions: N, M: Convert insecure short-term employment contracts to standard employment, using legislation and by facilitating agreements between employers and labor unions. N: Give all in short-term contracts adequate access to, and full coverage of, welfare benefits which are now linked to standard employment, e.g. unemployment, sickness and parental benefits. N, M: Develop basic income and debt settlement schemes. N, M: Improved access to rental apartments and home mortgage. N, M: Requirement of standard full-time employment as a norm in public procurement. N, M: Require labour standards in public procurement and improve monitoring of the requirements set. M: Increase predictability in workplace time management. N, M: Improve opportunities to join and organize in trade unions.
4. Good school climate could be protective for later mental health symptoms	Goals: Good school environment Suggested interventions: N: Evidence-based interventions, including reducing class size, and improving teacher quality, especially in disadvantaged areas should be a priority [17]. N: Improve the supervision of the schools’ compliance with the Work Environment Act. M: Strengthen the prevention of bullying at school, including measures against cyberbullying. N, M: Strengthen the legislation and control of availability to school health care. N, M: Improved teacher education and demands to employ graduated teachers. Improved working conditions for teachers. N, M: More resources are needed to prevent pupils from leaving compulsory school with incomplete grades. N: Abandon the school market to create more equity in education with equal access to highly educated teachers and with equal possibilities to receive high grades.
5. Poor working conditions can cause poor mental health.	Goals: Improve psychosocial and other working conditions and rights. Suggested interventions: N, M: Promote and seek possibilities to increase job control, decrease job-strain, enhance effort-reward balance, and organisational justice at the workplace, especially for low-skilled workers. Adapt to local conditions with involvement of employees and managerial support. M: Reduce strenuous work demands when feasible. Enhance job control for remaining work tasks. Avoid work exceeding 30% of physical capacity. N, M: Promote a better match between job demands and the individual work capacity by facilitating continued education and possibilities to test new jobs in adult age and reduced working hours. N, M: Improve possibilities to work part-time at higher age without losing employment/income protection, especially for those with low-medium education and low job control or high physical work demands.
ADVERSITIES	
6. Material and social adversities can cause mental ill-health.	Goal: More equal living conditions (educational achievement in relation to own capacity and labour market opportunities). Suggested interventions: All policies should contribute to counteract marginalisation and loss of resources as well as to help individuals to gain control over life. The following suggestions were put forward by the Swedish Commission on Equity in Health: [18] 1. Changes in tax policy have with a redistributive effect, for example increased progressivity, reintroduced wealth taxation and equal taxation of different forms of housing. Today, rental apartments are taxed more heavily than own houses and condominiums. 2. A changed fiscal policy where the current budget ceiling is lifted, and funds are released for increased investment in human capital in the form of better education and health promotion measures. 3. Increased financial transfers to groups with insufficient income, such as the disabled, early retirees and families with children. 4. Increased regional and municipal tax equity with the aim of achieving equivalent healthcare, school and community services in all parts of the country.
GENDER RELATIONS	
7. Gender inequities in working life are related to poor mental health.	Goals: Increase gender equity and rights in working life. Suggested interventions: N, M: The tendency to create parttime contracts in women-dominated sectors should be counteracted through construction of permanent contracts with full-time employment and coherent working time. N, M: Secure supply of skilled workers in the healthcare sector by offering good working conditions, sufficiently high income and on-job upskilling. N: Diminish the gender gap in salaries, a new EU Directive requires that reporting is combined with penalties. Decrease women’s vulnerability on the labour market by considering possible effects on women’s health and bargaining power when changing access to the welfare system (N), when planning public transport (M) and opening hours and fees for childcare (M). N, M: Political demands for reduced working hours with full salary in strenuous occupations. N, M: Increase availability of high-quality childcare and free school lunches up through secondary school.
8. Gender inequities in private life are related to poor mental health.	Goals: Increase gender equity and rights in society as well as in the family sphere. Suggested interventions: N: Concretise and implement the gender equity policy aim that everyone, regardless of gender, has equal rights and opportunities. N: Individualize the entire parental insurance to increase men’s outtake. N: Zero tolerance of all sorts of violence against women. Prevent with laws, transformed gender norms, and increased knowledge of women’s human rights. N: Provide services for survivors, including helplines, safe housing for women and children who are victims of abuse
SOCIAL SUPPORT	
9 Poor social support is detrimental to mental health over life. Being member of clubs/associations improve mental health.	Goal: Increase the level and improve the quality of social support. Suggested interventions: M: Support civil society, both in terms of financial resources as well as concerning infrastructure, e.g. by creating meeting spaces in the local community. M: Involve civic society in local community processes, like parental-school collaboration, or through local sports, culture or other leisure activities. M: Involve older people in supporting younger people to enhance intergenerational transfer of social resources. M: Develop participatory methods for physical planning e.g. in development of new and renovation of old building environments, or by restructuring of local transport systems and use of urban green spaces. M: Establish ‘time banking’ systems which can facilitate the exchange of services between individuals in a neighborhood, e.g. baby-sitting, shopping assistance, or various home repair or gardening services. Goals: Increase the proportion of young people involved in social clubs and associations. Suggested interventions: N, M: Political measures to increase the accessibility (both financially and physically) of sports clubs, cultural centres (including music classes) and other associations. This is especially important for young people.
NEIGHBOURHOOD	
10. Disadvantaged neighbourhood seem to have a weak but constant negative impact on mental health over life	Goals: Counteract residential segregation by mixed types of housing and ownership. Avoid accommodating those not able to find housing on the regular market in a way that aggravate segregation. Give priority to improved housing and neighbourhood qualities including safety in currently disadvantaged areas. Suggested interventions: M: Map aggregated social and environmental adversities N, M: Direct local area physical planning and renovation towards environmental equity. Include compensatory aspects in the planning of outdoor and indoor space to promote social networks and social participation, and access to public services for those who have limited mobility due to e.g. health, family or financial constraints. N, M: In physical planning, apply a systematic approach to avoid a disproportionate load of infrastructure, and housing of the homeless/recent immigrants in neighbourhoods with aggregated adversities/poor neighbourhood quality. N, M: Policies for increased access to green and blue areas for all segments of the population. M: Enhance the voice of local communities. When upgrading/restructuring disadvantaged areas, use a participatory approach to explore possibilities to promote local business, employment, social participation and communication between residents and decision-makers. N: Create special subsidized mortgage and savings schemes to enable financially challenged people to buy their own homes. N, M: Develop national and local strategies to ensure adequate housing for all strata of the population. Consider public housing construction and investment subsidies as means to achieve this. N: Create policies that reduce inequities between different forms of housing arrangements. N: Address the present inequities effects of tax reduction schemes for repair and renovation of flats and houses and regarding household services.

Of our ten key themes, three were obviously related to equity in relation to social class (“Material and social adversities …”, ”Poor working conditions…” and “Disadvantaged neighbourhood .”) while two were clearly gender-related (“Gender inequities” in working and in private life, respectively). For the five remaining key themes, references to equity dimensions have been added.

The following five domains were identified: working life (including school), social class relations, gender relations, social relationships, and residential neighbourhood.

## Results - Suggested policies for primary prevention of poor mental health

The following ten key themes were identified and the total number of original publications contributing to them is shown in brackets. One publication can contribute to several themes. (1) Unemployment can cause poor mental health (21 papers). (2) Active labour market programs may counteract poor mental health (3 papers). (3) Temporary employment is detrimental to mental health (12 papers). (4) Good school climate could be protective against later mental health symptoms (7 papers). (5) Poor working conditions are related to poor mental health (3 papers). (6) Material and social adversities can cause poor mental health (8 papers). (7) Gender inequities in working life are related to poor mental health (2 papers). (8) Gender inequities in private life are related to poor mental health (9 papers). (9) Poor social support is detrimental to mental health (9 papers). (10) Disadvantaged neighbourhood seem to have a weak but consistently negative impact on mental health (11 papers).

Below, the key themes have Arabic while the domains have Roman numerals.

### Working life

Until the deep recession of the 1990s, the studied cohort had lived in one of the most secure welfare states in the world. Around age 28, they met sharply increasing rates of unemployment in combination with increased job insecurity, manifested as an increase in uncertain job contracts.

1. Unemployment can cause poor health: with both direct and long-term effects.
  As unemployment is socially constructed it should, at least in theory, be possible to avoid. An obvious primary prevention strategy is thus to combat unemployment [19]. To create more jobs, several policies can be applied including reduction of working hours and overtime work as well as to improve the match between labour demand and supply through educational policies. A shift between taxes on work and capital has also been suggested [16].
  Secondary prevention includes policies that could diminish the negative effects of unemployment, ranging from economic compensation to social and psychological support [20]. Tertiary prevention could include rehabilitation and activities to keep labour skills updated are also important [21].
2. Active labour market programs (ALMP) can counteract poor health among those exposed to unemployment.
  Participating in ALMPs was beneficial for mental health among both teenagers and in older age groups [11]. Secondary prevention effects of ALMP include elimination of the negative effects of unemployment, ranging from economic compensation and restoring the time-structure of the day, to social and psychological support [21]. In addition, treatment studies (tertiary prevention) have shown positive effects [22].
3. Temporary employment contracts is detrimental to mental health.
  Short-term educated are most badly hit by the mental health consequences of temporary employment [11]. Primary prevention includes policies to secure employment contracts through legislation and facilitating agreements between employers and labour unions.
  Benefits currently tied to permanent jobs should also be offered to those with temporary jobs. Other possible policy options are basic income schemes, debt settlement schemes (where individuals are given the same rights as business enterprises to settle their debts and recreate a viable financial platform) [23] and improved access to credit for buying your home or by removing barriers for tenancy (like the requirement for long-term employment contracts or strict demands on income stability).
4. Good school climate could be protective for later mental health symptoms.
  Having good school climate was protective for childhood adversities in relation to adult health [24]. The goal of this key theme is to create a good school climate for all pupils. A good school climate was in the NoSCo studies defined as including decision making, good peer relations, satisfaction in school, meaningful learning, making use of interests and talents. Nowadays, the school environment of all pupils is protected by law and systematic work environment management is required. Despite the legislations, mental health problems in school age have increased [5–7] and the changes of the Swedish school system has been repeatedly criticized for deteriorating results across several decades and increasingly associated with parental educational level [25]. Thus, evidence-based interventions, including reducing class size, and improving teacher qualifications, especially in disadvantaged areas, should be a priority [17]. In addition, access to school health services (which all pupils are entitled to) should be improved. The deteriorated school results are further elaborated in the Discussion.
  Schools need educated teachers and a good school environment not only for pupils but also for teachers. In addition, there is a need for more resources to prevent pupils from leaving compulsory school with incomplete grades, as lack of education markedly increases the risk of being exposed to a weak labour market attachment (LMA) during life.
5. Poor working conditions can cause poor mental health.
  Those working in temporary employment had lower decision latitude [11], which per se is related to mental ill-health. In addition, physically heavy jobs had a strong negative impact on women’s mental health [11].
  The contribution from physically strenuous work to inequities in health may be alleviated by risk assessment and primary prevention, e.g. implementing “safety-by-design” production processes, risk assessment mitigating high risks (including e.g. increased staffing and job control in home care), and access to occupational health services with ergonomic expertise for prevention on both individual and group levels. Collaboration between employers and occupational health services for those needing adjustment of work tasks or help to qualify for a less physically demanding job is increasingly important as retirement age is raised while gains in health are virtually absent for those with the lowest levels of income or education [26]. Government policies should ascertain a match between need and access to such services.
  In general, a good work environment does not only reduce the risk of work-related poor health but can also be anticipated to be more inclusive for people with disabilities by controlling excessive demands and adjusting them to individual capacity.

### Adversities

6. Material and social adversities can cause mental ill-health.
  Since the Marmot report [27], the importance of structurally oriented public health initiatives addressing the social drivers of adversities has been consistently emphasized. A systematic review shows that policies in high-income countries that improve the generosity of social security benefits can improve mental health [28].
  Several suggestions [18, 29] have been put forward aiming at more equal living conditions to reduce inequities in health. A common focus has been on financial redistribution through taxes, transfers and fiscal policies. Thus, financial transfers can have significant effects on mental health inequities across different recipient groups.

### Gender inequities

7. Poor mental health is related to gender inequities in working life.
  Analyses of gender inequities at the workplace showed that women in traditionally gender unequal workplaces (measured with a workplace gendered index of education, salaries, sick leave, etc.) had most psychological distress. The lowest levels of psychological distress were found in the most gender equal workplaces, for both men and women. Thus, workplace gender equality was beneficial for both genders [11].
  Regarding salary inequities, a new EU Directive (2023/970) for wage transparency will require changes from both regulators and partners and requires that reporting is combined with penalties [30]. Also, eliminating the gender pay gap requires addressing its underlying causes: unequally distributed unpaid work which in turn is related to women’s higher rates of part-time work and career breaks [31].
8. Poor mental health is related to gender inequities in private life.
  The negative health effects of gender inequities in private life (like unequal division of household work) may be counteracted by policies to increase equity in unpaid work, such as equal division of paid parental leave.
  Men’s physical, psychological, and sexualised violence against women is a major public health problem [32] which despite strong policies worldwide [33] (including an EU Directive [34]) to end men’s violence against women continuous to be in need for more preventive efforts to urgently reduce such violence [35].

### Social support

9. Poor social support is detrimental to mental health.
  The goal of this key theme is to increase the level, and improve the quality, of social support. Policy interventions could include involvement of voluntary organizations in local community processes, like parental-school collaboration, or local sports, culture and other leisure activities which may counteract marginalisation. Policies could also aim at developing more participatory methods for urban planning, e.g. in the development of new and renovation of old built environments, restructuring of local transport systems and urban green spaces. Also, ‘time banking’ systems may facilitate exchange of services between individuals in the neighbourhood, e.g. baby-sitting, shopping assistance, or various home repair or gardening services [36].
  Activity in clubs at young ages was shown to improve mental health. In addition, it protects against the negative effects of childhood adversities on adult health [12]. Providing resources, including meeting spaces, to the voluntary sector could increase the access (both financially and geographically) for vulnerable groups to supportive environments such as sports clubs, cultural centres and other associations.

### Neighbourhoods

10. Disadvantaged neighbourhoods have a weak but consistently negative impact on mental health.
  Primary prevention strategies could counteract residential segregation by providing mixed types of housing and by avoiding accommodating disadvantaged groups in negatively segregated areas. Mapping of aggregated social and environmental adversities by neighbourhood (e.g. using geographical information systems) is a means to identify priority areas for interventions. Local area urban planning and renovation should be directed towards environmental equity, using a place-based and participatory approach [37]. In priority areas, compensatory measures should be considered in the planning of public indoor and outdoor spaces to promote social networking and participation.
  National and local strategies should ensure adequate housing for all, independent of socioeconomic resources. In Sweden in 2024, overcrowding in the population with the lowest income quintile was more common (45.5%) than in the European Union as a whole (27 countries, 32.1%), despite a lower overall rate of overcrowding (15.7% and 18.2%, respectively) [38]. This highlights the need to provide affordable housing.
  Inequities in e.g. taxation regarding different types of housing should be addressed. Subsidized financing of flats to accommodate large families should be considered, as should special mortgage and savings schemes to enable those with low incomes to buy their own homes.

## Discussion

In this paper we have performed qualitative analyses of cohort-based evidence, with a broad life-course approach, and synthesised actionable results in ten themes, for which we have identified evidence-based policies for improved and equitable mental health from adolescence until midlife.

Working life is a major domain in our results and our findings about the mental health consequences of unemployment have been verified in contexts around the world [39]. The mental health consequences may be buffered by education in terms of university exams as well as by social relations and state welfare support [40].

In addition, there is also evidence from the US that employment insurance reduced stress among short-term educated unemployed [41] and, mainly from European countries, that ALMPs improved mental health [42].

In theme 5 we identified that poor working conditions are related to poor mental health. Poor psychosocial working conditions have consistently been related to the onset of depression, and to sick leave due to mental disorders [43]. According to the Swedish Work Environment Act, the employer is responsible for assessing and mitigating risks regarding the physical, as well as the social and organisational, work environment. Measures enhancing job control, involving the employees in creating solutions, and with full support from high levels in the organization have been shown to be successful [43]. For example, moving away from micro-managed time schedules for elderly care workers and permitting the employees and their clients to agree on the priorities has been reported to result in higher mutual satisfaction, enhanced job discretion and recognition within the organization for the employees [44].

A focus on low-skilled and marginalised workers, usually facing both the poorest job conditions and the highest risks, has been recommended, with measures including primary prevention (improving working conditions), increased awareness of mental health problems, and facilitating return-to-work after sickness absence and unemployment [43]. Workers born outside Sweden (and especially outside Europe) are over-represented in such jobs.

We found that gender inequities in working life are related to poor mental health. Today, women in the Nordic countries participate to a high degree in paid work, but within an extremely gender segregated labour market, both vertically and horizontally, which leads to inequities in health due to unequal power, influence, career opportunities, salary and work environment. Men and women working in women-dominated workplaces have for example the highest sick-leave which in turn can be related to the deteriorating psychosocial work environment in women-dominated workplaces [45–47].

The welfare sector needs increased staffing now and in coming years. The most significant obstacles to attract more men are low wages, limited career paths, and unfavourable organisational conditions, gender norms, and occupational gender labelling. Addressing these problems requires a comprehensive approach to improve the working conditions and at the same time combating stereotypical gender norms in education and workplaces.

Future research could implement an intersectional perspective which goes beyond additive analyses of for example gender and socioeconomic conditions to study complex intersections between the major factors which potentially influence health and ensure that gendered power relations and social context are included [48].

Our policy suggestions need to be discussed in relation to the political development in Sweden since the cohort was young during the 1980s. A Swedish government investigation [18] states that:

1. Economic inequity has increased because transfers to groups outside the labour market have not kept pace with the general income trend.
2. Regional inequity has increased, which has particularly affected regions outside the metropolitan areas.
3. Due to immigration and increased segregation in schools, inequity in education has increased and young people with low education have substantial difficulties entering the labour market.
4. The integration policy has been insufficient to meet the needs of immigrants with low education, and of their children.
5. Too little housing construction has led to an increased housing shortage, which has affected mainly young people with low incomes and contributed to increased segregation.

These political developments have led to increased gaps between those who participate in working life and those who do not enter or are excluded from the labour market. The goal of full employment has lost its central role in Swedish financial policy since the 1990s in favour of inflation targets and budget constraints. The policy of improving the qualifications of those in search of work has been substituted by coaching activities to increase their intensity of job searching. Active programs have been replaced by less effective, more expensive [49] and more defensive programs with the main goal to maximise the supply of manpower [50]. In fixed monetary value, income from employment has risen by 70%, while income from transfers to groups outside working life have remained unchanged since the 1980s [51]. In addition, the gap between individuals with financial assets and those without, largely supersedes income inequities [3]. Inequities in capital income, is therefore the main explanation for the increasing Gini-coefficient regarding income in Sweden.

All these changes are likely to increase the inequities in mental health, especially among young people and among those living outside the metropolitan areas. In addition, attention must be drawn to the possible negative impact of social media on mental health [52], especially of young people. UNICEF collaborates with major social media (e.g. Instagram, TikTok, Facebook/Meta) to provide information, safety guidance, and joint initiatives on online safety and cyberbullying. Even though social media activities mostly focus on the individual child/parent they also to some extent address the structure and function of the platforms.

The effects of neoliberalism on health inequities in Sweden have been studied in relation to different welfare policy domains. The strongest effects were found for unemployment benefits [53], which may be related to the stigma of unemployment. In relation to our findings of the negative health effects of weak LMA and financial insecurity as a major mechanism, increasing inequities in health may be expected. Restrictions in access to disability pension and changes in retirement policies have also been shown to increase educational inequities in exit pathways from the labour market, and more so among women, with more negative financial consequences for those already in a vulnerable position [54]. For many decades, a relatively equal level was guaranteed in Swedish schools, but significant differences in quality now exist. Contributing factors to the increased segregation are policies including decentralisation as well as introduction of school choice, privatisation and profit-making free schools with rights of free establishment [18]. Private schools are paid the same amount per pupil as public schools are, independently of real costs. This favours private schools which tend to establish themselves in affluent, high-educated areas with “less costly” pupils. They also compete in the ‘school-choice’ system by more lenient grading standards, as they tend to award unjustified higher grades than public schools [25]. Public schools are thus caught in a harder financial situation as they (with unchanged payment) must take the higher costs for pupils with extra needs. Lower resources in public school therefore increase the risk that their pupils get less qualified education which in turn is related to poorer mental health.

### On the methods

#### Strength and limitations

The main limitation is the use of one relatively small database. However, even though the cohort originates from one municipality, it has been shown to be representative of the country. The modest sample size makes it possible to keep contact with all participants during the follow-ups, including those in NEET (not in education, employment or training) and to reach an extraordinary response rate over three decades.

We used qualitative methods to summarise and synthesise earlier publications. Summative content analysis is a widely used and well-established qualitative method to analyse texts, like the two meta-syntheses and the papers they are based on in this study. To achieve trustworthiness, all original publications were kept, except three cross-sectional studies. No other exclusions were made. The coding of all material was made in a systematic and careful way by several of the authors. To minimise bias, the development of themes has been carefully described.

A qualitative design does not make it possible to analyse the effect size or the statistical significance of the suggested policies. But our research has been performed on a cohort with extremely high participation rate from adolescence until adult age. The context of the cohort differs today, mainly in relation to immigration and in the dismantling of the Swedish welfare system. However, most determinants of poor mental health which we have identified above tend to be overrepresented by foreign-born individuals [55] which would make our suggestions even more pertinent in populations of today.

The main strength of the paper is our extensive research on social determinants of mental health on which the suggested policies are based. We are aware that researchers from other disciplines like genetics or economy could have suggested other policies.

We have presented the Swedish case, but there are many similarities with other high-income countries. For example, European researchers have presented a framework of questions that may provide a tool for a dialogue between researchers and policymakers [29]. They conclude that even though all answers are not yet available, they believe that discussing the questions in relation to a specific population in a specific political context will be fruitful to inform policies on health inequities. We believe that our Swedish experience can be informative also in a wider global context, regarding identifying actionable results and link them to policy contexts (although policies always must be assessed in relation to the special case), and because the rapid growth of inequities in income and health has showed the impact of adverse policy shifts.

Our suggested measures are well in line with the Swedish determinant-based public health policy with the objective to eliminate avoidable health inequities within a generation. The implementation of the proposals would be greatly facilitated if, like in Norway, there was a Public Health Act, which clearly defined the responsibility of municipalities and other actors for implementing and following up the public health policy.

A recent follow-up study of the Final Report of the WHO Commission of Social Inequities in Health concluded that improvement in equity in most countries has been slow, with tendencies towards increasing inequities in health [55]. The authors recommend several diverse action areas including addressing economic inequality as well as strengthened focus on, and monitoring of, social determinants of health in different policy areas.

Although the specific examples we have provided are shaped by the Swedish context, they illustrate a broader trend in which public welfare services are increasingly reorganised along market-oriented principles. This shift often results in service allocation becoming more closely tied to individuals’ ability to pay, while access for those with the greatest needs is constrained through reduced public financing and governance mechanisms that disproportionately disadvantage socioeconomically vulnerable groups. Hence, the Swedish case may offer insights into more general patterns observed in countries implementing neoliberal policy reforms.

## Conclusions

Our qualitative analyses of cohort data, with a broad life-course approach, synthesised actionable results, from which we identified evidence-based policies for improved and equitable mental health from adolescence until midlife. Our suggested policies can contribute to realise a “Health in all policies strategy”, as suggested by the Helsinki statement. We encourage further systematisation of evidence-based public health policies in other contexts, including the ongoing marketisation and underfunding of the welfare sector in high-income countries.

Our identified policies are related to several of the Sustainable Development Goals including Good Health and Well-being, Quality Education, No poverty, Gender Equality and Decent Work, Reduced Inequality as well as Sustainable Cities and Communities. Funding that supports health equities and allocations by needs should be regarded as investments for sustainability.

## Data Availability

Data can be retrieved from two meta-analyses which are freely available as open-access publications.

## Abbreviations

NoSCo: Northern Swedish Cohort
LMA: Labour market attachment
ALMP: Active labour market programs
NEET: Not in education, employment or training
